# Social perception of robots is shaped by beliefs about their minds

**DOI:** 10.1038/s41598-024-53187-w

**Published:** 2024-03-05

**Authors:** Ali Momen, Kurt Hugenberg, Eva Wiese

**Affiliations:** 1 United States Air Force Academy, Colorado Springs, CO USA; 2 George Mason University, Fairfax, VA USA; 3 Indiana University, Bloomington, IA USA; 4 Berlin Institute of Technology, Berlin, Germany

**Keywords:** Human behaviour, Mechanical engineering

## Abstract

Roboticists often imbue robots with human-like physical features to increase the likelihood that they are afforded benefits known to be associated with anthropomorphism. Similarly, deepfakes often employ computer-generated human faces to attempt to create convincing simulacra of actual humans. In the present work, we investigate whether perceivers’ higher-order beliefs about faces (i.e., whether they represent actual people or android robots) modulate the extent to which perceivers deploy face-typical processing for social stimuli. Past work has shown that perceivers’ recognition performance is more impacted by the inversion of faces than objects, thus highlighting that faces are processed holistically (i.e., as *Gestalt*), whereas objects engage feature-based processing. Here, we use an inversion task to examine whether face-typical processing is attenuated when actual human faces are labeled as non-human (i.e., android robot). This allows us to employ a task shown to be differentially sensitive to social (i.e., faces) and non-social (i.e., objects) stimuli while also randomly assigning face stimuli to seem real or fake. The results show smaller inversion effects when face stimuli were believed to represent android robots compared to when they were believed to represent humans. This suggests that robots strongly resembling humans may still fail to be perceived as “social” due pre-existing beliefs about their mechanistic nature. Theoretical and practical implications of this research are discussed.

## Introduction

In recent years, deepfakes—artificial intelligence (AI) generated images often involving celebrities or politicians—have gone viral on the internet, enabled by their hyper-photorealistic appearance. Such AI-produced content is powerful and ranges from synthesizing individual’s speech and voice^1^, to creating images of fictional people^2^, to swapping a person’s identity with another or altering what they are saying in a video^3^. Besides offering exciting opportunities, such technologies are widely recognized as a pressing threat to the believability of perceptual information^4^ and many cases of misuse have been reported to date (e.g., deepfakes of Donald Trump being arrested; fake images of the pope in a puffer jacket,^5^). Artificially recreating human appearance to an extent that it is not easily distinguishable from actual humans is also a major topic of interest in robotics, where androids very convincingly emulate their human counterparts (e.g., the android robot Sophia;^6^).

Faces are most often the targets of such manipulation because they are a rich source of social information, including cues to identity and internal states^7–10^, and critically determine the effectiveness and believability of supposedly "human" content^11,12^. Significantly, it has been consistently shown people are particularly attuned to a face’s orientation, with upright faces being recognized more readily than inverted faces. Since the seminal work by Yin in 1969, studies have shown that turning a face upside down interferes with our ability to process and recognize them far more than when the same is done to objects.^13–15^. Multiple theoretical accounts have been made for the face inversion effect, with some arguing that faces are special and processed in a qualitatively different manner than non-social objects^16^; others argue that faces are not special but are rather processed in quantitatively different manners than non-face stimuli^17,18^. However, although there is some debate regarding the “special status” of faces, there is consensus that inversion does disrupt face-like processing more strongly than the processing of non-face objects^13^. This effect on face recognition occurs immediately after a face stimulus is presented and can be measured by activity in the N170 event-related potential—a neural indicator of face perception. It also triggers heightened fMRI activity in brain regions that are typically involved in recognizing objects^19,20^. Indeed, face processing is subserved by a core network that concerns the bottom-up processing of facial features (i.e., eyes, nose, mouth), as well as the spatial relations between these features: the inferior occipital gyrus or occipital face area (OFA) encodes isolated facial features (e.g., eye color), whereas the lateral fusiform gyrus (or fusiform face area; FFA) supports the configural processing of human faces and represents unchangeable facial information related to an individual’s identity^21^. Changeable face features, on the other hand, such as changes in gaze direction to signal an action intention or facial expression to signal an emotional state, are processed by the posterior superior temporal sulcus (pSTS; see^22^ for a review).

But how strongly will hyper-photorealistic yet synthetically generated face stimuli, such as images of deepfakes or androids, trigger face-typical processing^23–25^? Hyper-photorealistic depictions are easy to produce with deep-learning models, such as Generative Adversarial Networks (GANs), which pit a generator and a discriminator neural network against each other so that—over multiple iterations—the generator learns to synthesize increasingly realistic face stimuli until the discriminator is unable to distinguish them from real faces^11^; similar results can be obtained using diffusion models^26^. The generated images are often so visually convincing^27^ that even sophisticated algorithms cannot reliably distinguish them from real human faces^28–31^. Indeed, whereas early deepfakes were still detectable by humans^32–34^,

the synthetic faces now being created are so lifelike that they are often indistinguishable from genuine human faces^11,23,24^. Due to their photorealistic visual similarity to human faces, one would expect that deepfakes activate the core face perception network in a similar manner as human faces. However, both behavioral and electrophysiological studies indicate that deepfake images are processed differently than human faces. ERPs like the N170^35^ or steady-state visually evoked potentials (SSVEPs;^36^), for instance, appear sensitive to actual versus artificially generated faces. Furthermore, computer-generated faces do not always elicit face-processing phenomena typical of real faces (e.g., “other race effect”;^37^). Thus, although highly realistic to the point of near indistinguishability from actual human faces, computer-generated face stimuli can differ in their manner of processing.

How might we understand why even hyper-realistic synthetic faces elicit a different manner of processing than actual human faces? We believe that this may be due to the core face network receiving top-down input from an extended network of other brain areas involved in social cognition (see^22,38^). This suggests that face perception may be the product of both perceptual characteristics of the face stimuli themselves and higher-order social cognitive influences reflecting perceivers’ motives and beliefs^39,40^. Thus, even when synthetic face stimuli are perceptually indistinguishable from real human faces, they might still not be processed in the same way as human faces when perceivers know they are artificial. Perhaps simply believing a face to be artificial can influence the extent to which it is processed in a face-like manner. The most conservative test of this hypothesis would be to take actual human faces and make participants believe—through a cover story—that they are not human. Would such a top-down manipulation of beliefs be sufficient to attenuate face-typical processing, and as a consequence lead to a reduced face inversion effect?

This question is highly relevant not just to understanding the processing involved with deepfakes and androids, but also applies to the perception of human faces themselves. In most traditional face perception studies, the perception of a stimulus (like a face) cannot easily be separated from people’s beliefs about that stimulus (for instance, believing it represents a human). Consider, for instance, Yin’s classical work^14,15^ on the face inversion effect, where perceivers saw human faces and non-face objects, such as houses: they almost certainly thought that the faces represented actual people, and the houses real objects. However, with hyper-realistic face stimuli like deepfakes^11^ or highly human-like robots like androids^41^, we live in an era where percepts and beliefs are not necessarily conflated. This creates the possibility of dissociating bottom-up effects associated with the percepts of human faces from top-down effects associated with beliefs about human faces, and allows us (i) to examine to what extent face processing is cognitively penetrable^42,43^, and (ii) whether beliefs about a face’s essential humanness influence the manner in which it is processed.

Indeed, there is evidence in the literature suggesting that manipulating perceivers’ beliefs about faces can modulate face processing^44^. For instance, when the same face is believed to belong to an ingroup (i.e., fellow university students), it is afforded stronger configural processing than when the face is believed to belong to a social outgroup (i.e., a competing university^45^). Similarly, a stronger N170 component was found when face stimuli were categorized as belonging to the participants’ own social group versus an outgroup^46^. Beliefs about a target’s moral behavior can also modulate face processing. Fincher and Tetlock^47^, for instance, found that faces of targets believed to be engaged in immoral or inhumane behavior elicit less face-like processing across multiple measures (see^48^). Taken together, this suggests that beliefs regarding the human nature of faces can modulate the extent to which they are processed in a face-typical manner. What these experiments cannot answer, however, is to what extent the mere belief that a human face may represent an object—namely an artificially generated face or an android robot—may disrupt face-typical processing.

### Current research

We investigate the extent to which the face inversion effect—one of the longest standing and best replicated effects in face processing—depends on participants' beliefs that apparent human faces represent actual human or non-human agents (i.e., androids with hyper-realistic human-like appearance). To do so, we manipulate beliefs about images of actual human faces via instruction while holding the percept of faces constant: participants were led to believe they would see the faces of actual humans (labeled as *human*) or the faces of highly human-like robots (labeled as *android*); in actuality, all images depicted real humans. If merely believing that a face represents a human versus a non-human agent modulates face-typical processing, the face inversion effects should be stronger in the human than the android condition. If true, this would suggest that the face inversion effect is driven, at least in part, by beliefs about the humanness of the face stimulus itself.

The current research adds to the previous literature on face perception in multiple ways. First, past work has focused on how the perceptual characteristics of faces influence face-like processing of non-human faces—such as those of robots—in a bottom-up manner. For example, Momen, Hugenberg, and Wiese (2022) had participants complete a face inversion task with human and humanoid robot faces, finding that robot stimuli elicited smaller inversion effects than human stimuli; it was also found that robot faces that physically resembled human faces (i.e., they contained more human-like facial features) were processed in a more face-like manner than were robots that appeared less human-like^49^. The present study builds upon this work in multiple ways. First, by keeping the bottom-up features of a stimulus constant but manipulating only participants' beliefs about the human nature of the stimulus. Second, although past work has already demonstrated that beliefs regarding one's social group status or morality can exert a top-down influence on face processing^45,46^, they cannot answer the question of whether beliefs regarding an agent's humanness also modulate face processing.

## Methods and materials

### Participants

A power analysis conducted with the effect size (n^2^ = 0.07) of a previous experiment utilizing the inversion task in a within-subjects design (^50^; Experiment 2) indicated a sample size of 86 would yield more than 95% power. Given that we anticipated removing participants due to poor performance and/or failing the manipulation check at the end of the experiment (i.e., *Did you believe the android faces were creations of a robot designer we are collaborating with*?), we conservatively oversampled.

233 participants completed the study via Amazon’s Mechanical Turk for pay. Of these, 44 participants were removed due to poor performance in the face recognition task (d’ ≤ 0), and 99 were removed due to failing the manipulation check at the end of the experiment. This resulted in a final sample size of 90 participants (M_age_ = 32.90 years; Range = 18–68 years; 47 females). Filtered participants accounted for 57.10% of our data. Informed consent was obtained from all participants, and all procedures were approved by the Office of Research Integrity and Assurance (ORIA) at George Mason University (GMU). The experiment was performed in accordance with relevant guidelines and regulations.

### Apparatus

The experiment was run using the Inquisit 5^51^ platform online, which allows collection of behavioral data remotely over the web via participant keystroke. Participants completed the experiment locally on their computer after downloading the software. Attributes like screen size, keyboard or refresh rate depended on participant's individual computers and were not controlled.

### Stimuli

**Figure 1:**
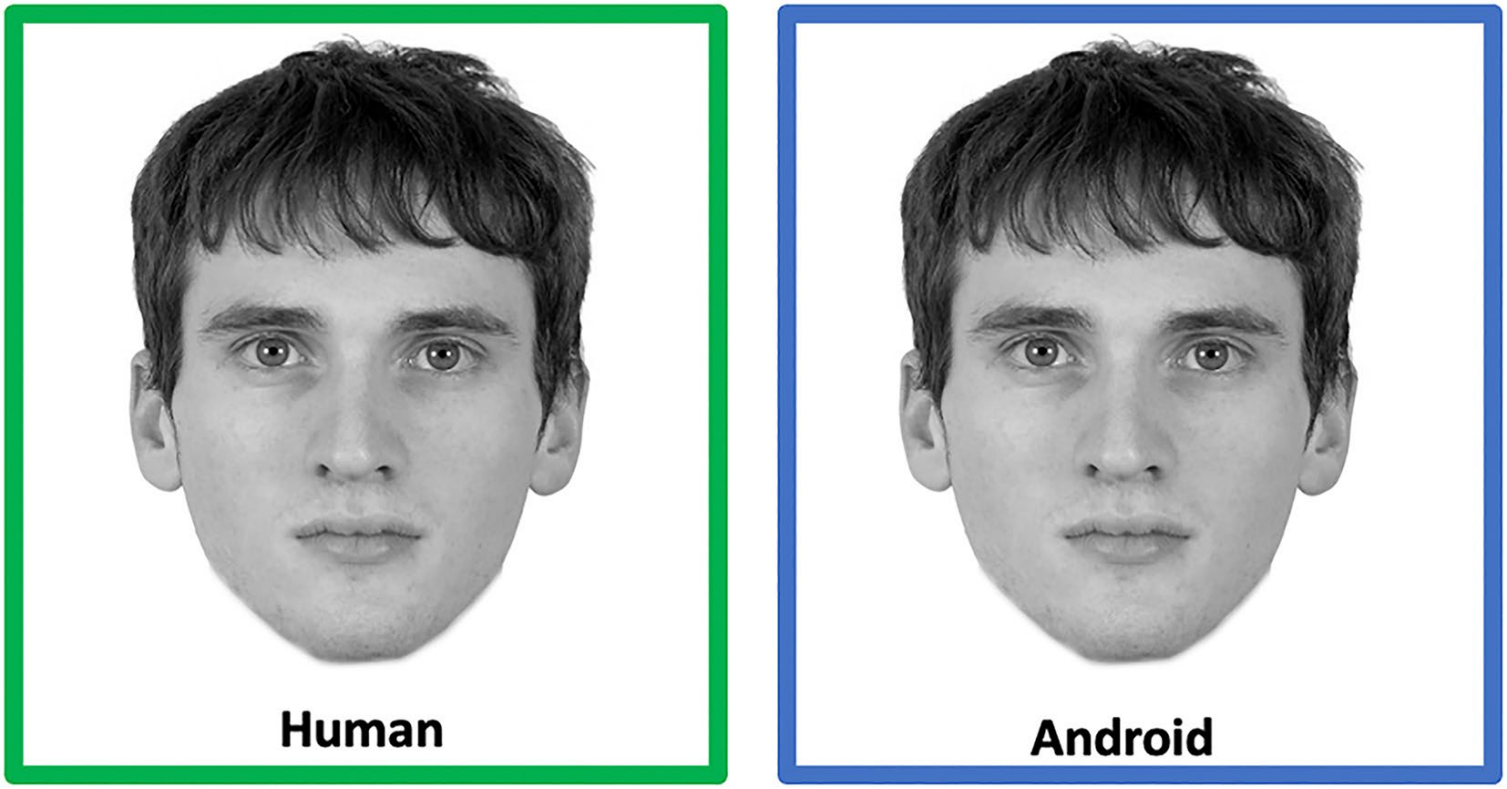
Example face stimuli. For each participant, faces were presented in the human or android condition in a randomized fashion. Each face was presented together with a label and a colored frame indicating the experimental condition: faces in the human condition were presented with the label “human” in a green frame; faces in the android condition were presented with the label “android” in a blue frame.

The sample of faces consisted of 80 White human male faces obtained from the Chicago Face Database^52^. All faces were converted to greyscale and presented on a white background that measured 768 × 768 pixels. The longest point, from the top of the head to the bottom of the chin, nearly touched both the top and bottom edges of the 768 × 768-pixel frame. Faces were also presented with labels and colored frames depending on their condition. Faces in the human condition were presented with an “human” label in a green frame. Faces in the android condition were presented with an “android” label in a blue frame; see Fig. 1. Consent to publish these stimuli images was obtained from the Chicago Face Database.

For each participant, the 80 face images were randomly categorized into distinct groups based on their designated condition (human vs. android), phase (learning vs. recognition) and orientation (upright vs. inverted). For the “*human*” condition, 40 randomly chosen images from the data base were allocated as follows: (1) *Presented in Learning Phase—Repeated Upright in Recognition Phase*: 10 images were randomly allocated to this category. These images were introduced upright during the learning phase and reappeared in upright orientation during the recognition phase. (2) *Presented in Learning Phase—Repeated Inverted in Recognition Phase:* Another 10 images were randomly assigned to this category. These images were presented upright in the learning phase and displayed invertedly during the recognition phase. (3) *Not Presented in Learning Phase*—*Distractor in Recognition Phase Upright*: Another 10 images were randomly assigned to this category. The images were exclusively used as distractors in the upright orientation during the recognition phase and were not part of the learning task. (4) *Not Presented in Learning Phase*—*Distractor in Recognition Phase Inverted*: Consisting of another 10 randomly assigned images, this category mirrored the previous one but with the images appearing invertedly during the recognition phase. The remaining 40 images followed the same distribution pattern as the human sets but were specifically used for the “*android*” condition. These images were similarly divided into repeated and distractor categories, with orientations corresponding to those in the human tasks. This random categorization into distinct groups was done before the beginning of the experiment separately for each participant to minimize the chances that any observed effects could be ascribed to the unique features of the faces.

### Procedure and task

Participants first gave informed consent and were then presented with the cover story: “*We are working with an android robot designer who designs robots nearly indistinguishable from humans. Here are some examples of his work*”. This was followed by images of android robots difficult to distinguish from humans (e.g.,^53^)*.*

Subsequently, participants completed the face inversion task. They were asked to memorize and recognize both human- and android-labeled faces. Agent type was blocked such that participants completed the learning and recognition task for one agent type (e.g., human) first before completing the learning and recognition task for the other agent type (e.g., android). Block order (android first vs. human first) was counter-balanced across participants with roughly half completing the android-labeled faces first (N = 44), and the other half completing the human-labeled faces first (N = 46).

**Figure 2:**
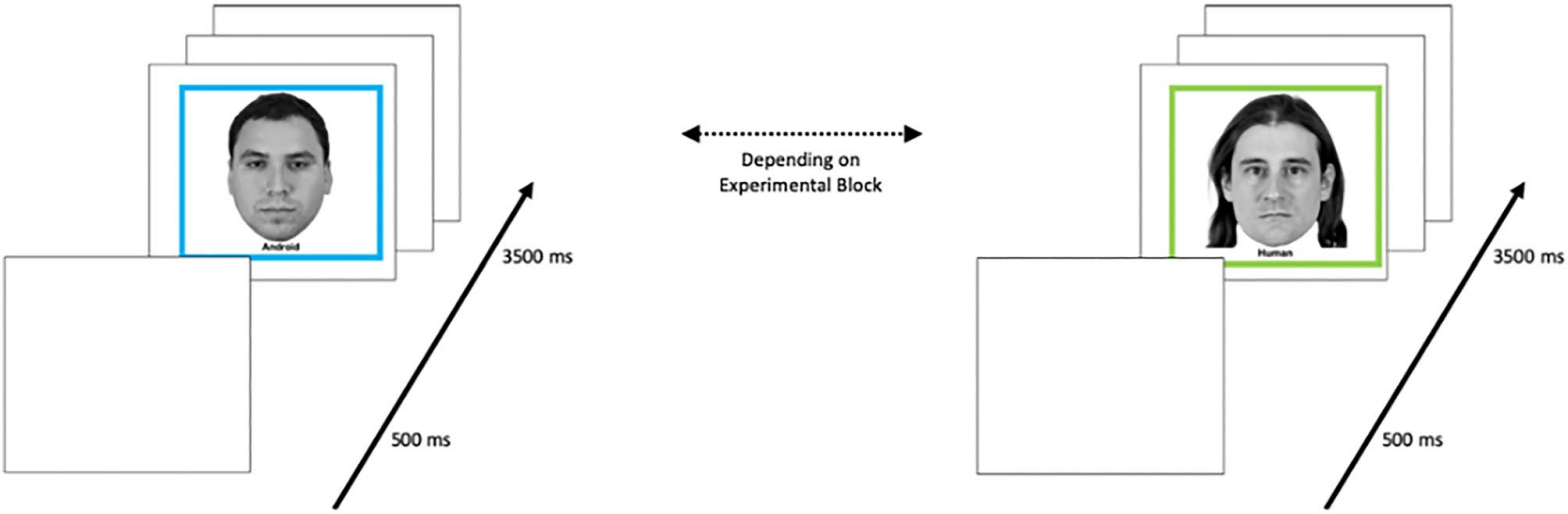
Learning phase of the inversion task. Participants were instructed that they would see twenty upright-presented human or android faces and were asked to attend to the faces for subsequent recognition. Participants passively viewed the 20 randomly selected faces in a randomized order for 3500 ms each, with an inter-trial interval (ITI) of 500 ms. Each participant saw both human-labeled and android-labeled faces. Agent type (human vs. android) was blocked, and block order was counterbalanced, such that half of the participants started the experiment with the android-labeled faces and ended the experiment with the human-labeled faces; the other half of the participants started the experiment with the human-labeled faces and ended the experiment with the android-labeled faces. Faces were randomly assigned to the “android” or “human” labels across participants (i.e., whether a given face was labeled as “android” or “human” randomly varied across participants).

During the learning phase, participants viewed 20 target faces for 3500 ms each, with an inter-trial interval (ITI) of 500 ms; see Fig. 2. The order of presentation was random. Faces in the human condition were presented with a “human” label in a green frame; faces in the android condition were presented with an “android” label in a blue frame.

**Figure 3:**
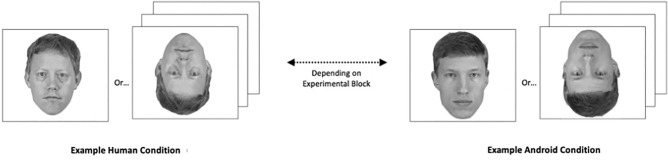
Recognition phase of the inversion task. At the beginning of the recognition phase, participants saw another series of faces, which included the 20 faces they had previously seen, interspersed with 20 new faces. These images were presented in random order without colored frames. Half of the new and half of the previously seen faces were presented upside down and participants were instructed that their task would be to report whether they had seen the face during the learning task.

During the recognition phase that followed, participants were presented again with the 20 faces they had seen during the learning phase, interspersed with 20 new faces. All face stimuli were presented in a random order, without original labels or colored frames. Half of the faces—previously seen and new ones—were inverted. Participants indicated if they recognized a face from the learning phase by pressing the ‘D’ key; if they did not recognize it, they were asked to press the ‘K’ key. Each trial in the recognition phase lasted until a response was given by the participant; see Fig. 3.

After completing both the android and human blocks, participants were thanked, for their participation, debriefed about the experiment, and compensated.

## Results

Of primary interest was whether there was a differential face inversion effect for faces labeled as “human” versus “android.” To assess this, we first calculated *hits* (i.e., familiar face correctly identified), *misses* (i.e., familiar face not correctly identified), *correct rejections* (i.e., unfamiliar face correctly rejected) and *false alarms* (i.e., unfamiliar face falsely identified) during the recognition phase. We then used these measures to calculate the signal detection parameter *sensitivity* (d’)—a measure of recognition that accounts for both hit rates and false alarms^54^. To account for 100% hit-rates and/or 0% false alarm rates, d’ scores were adjusted via a log-linear approach^54,55^.

Participants’ d’ scores were entered into a 2 (*Agent Label*: Human vs. Android) × 2 (O*rientation*: Upright vs. Inverted) repeated-measures ANOVA. If face-typical processing was attenuated by labeling a human face as android, a significant interaction effect between agent label and orientation would be expected, such that the difference in d’ between upright and inverted faces would be reduced for android-relative to human-labeled face stimuli.

**Figure 4:**
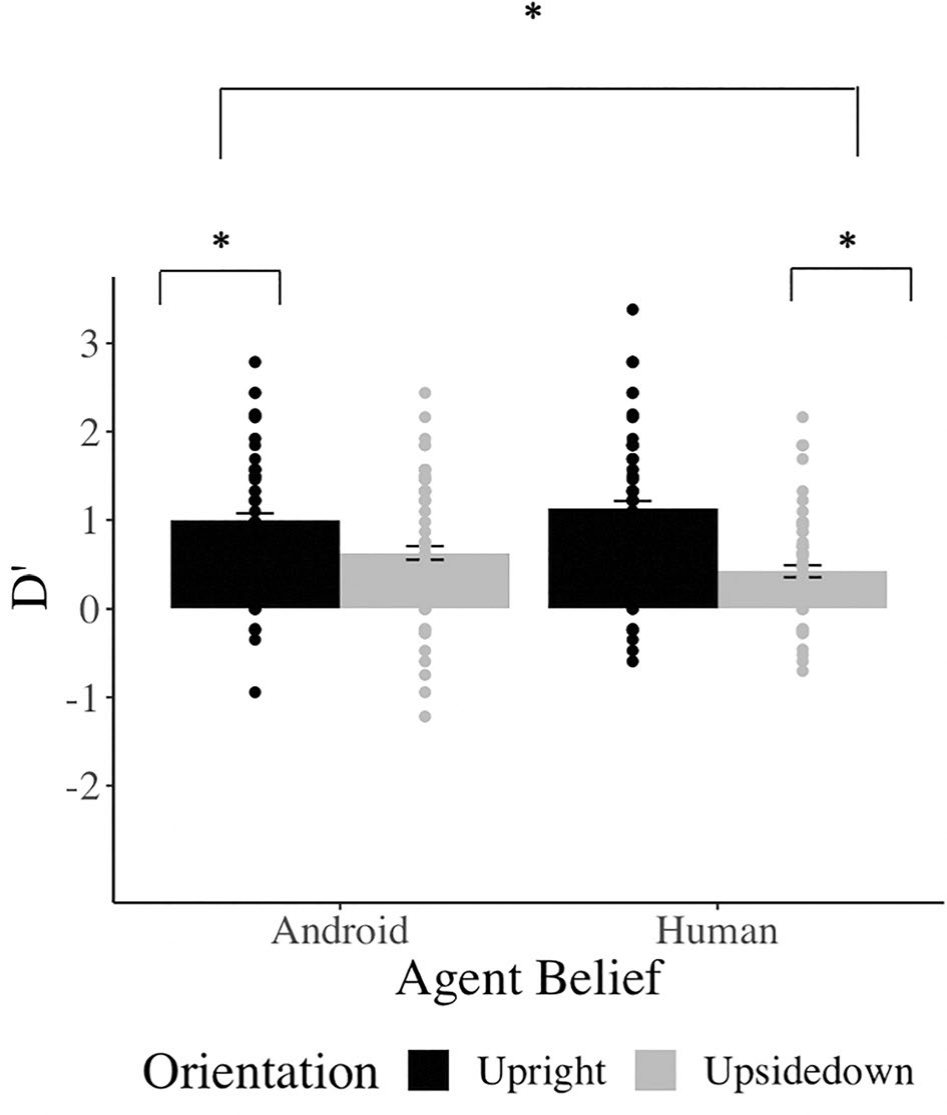
Main results: The 2 (agent label: human vs android) × 2 (orientation: upright vs inverted) ANOVA revealed a significant main effect of orientation, indicating a significant inversion effect across conditions. Importantly, the agent label × orientation interaction was also significant, indicating a stronger inversion effect when (perceptually identical) face stimuli were believed to represent human vs. android agents. Error bars represent 1 standard error above and below the mean.

The ANOVA revealed a main effect of *Orientation* (*F*(1,89) = 52.94, *p* < 0.001), such that participants had better recognition performance for upright (*M* = 1.06 *SD* = 0.52) compared to inverted (*M* = 0.52, *SD* = 0.68) faces; this represents the classic face inversion effect. There was no significant main effect for *Agent Label* (*F*(1,89) = 0.29, *p* = 0.593), indicating that the belief manipulation did not have an effect on participants' overall recognition performance. Most importantly, and in line with our hypothesis, the interaction effect between *Agent Label* and *Orientation* was significant (*F*(1,89) = 8.58, *p* = 0.004), such that there was a larger difference in recognition performance for faces believed to be human when presented upright (*M* = 1.12, *SD* = 0.83) vs. inverted (*M* = 0.42, *SD* = 0.61) than for faces believed to be android (upright: *M* = 0.99, *SD* = 0.77; inverted: *M* = 0.63, *SD* = 0.73); see Fig. 4. Although the inversion effect was significant for both android (*t*(89) = 3.74, *p* < 0.001, *d* = 0.48) and human conditions (*t*(89) = 7.93, *p* < 0.001, *d* = 0.97), the effect was more than twice as strong in the "human" than the "android" belief condition.

### Ancillary analyses

We also conducted two ancillary analyses. *First*, we explored whether explicit beliefs about the android nature of the images (i.e., participants who believed the manipulation) was required for the observed pattern of effects, or if the effects held even when including participants in the data analysis that did not explicitly believe the manipulation. For this purpose, we re-ran the original analysis where participants had been excluded from the dataset who did not believe the instruction manipulation, this time with the data of the 99 participants who had originally failed the manipulation check (total N = 189). The analysis showed that although there was a significant inversion effect across conditions (*Orientation*: F(1,188) = 192.33, p < 0.001), the *Orientation* × *Agent Label* interaction was no longer significant (F(1,188) = 1.91, p = 0.169). The main effect of *Agent Label* was not statistically significant; F(1,188) = 1.25, p = 0.265. This indicates that explicit beliefs about the human versus non-human nature of face stimuli are required for the observed modulation of face-typical processing.

*Second*, we investigated the criterion (c), which is the point at which individuals decide if a stimulus is familiar ('old') or unfamiliar ('new'). A positive criterion indicates a tendency to not recognize stimuli, suggesting a cautious approach, whereas a negative criterion suggests a tendency to recognize stimuli, indicating a more lenient approach. We submitted the criterion scores to a two-way ANOVA to evaluate the impact of orientation and agent label on participants’ decision thresholds. Neither the main effect of Orientation (F(1, 89) = 0.449, p = 0.504), nor the main effect of Agent Label (F(1, 89) = 3.015, p = 0.086) were significant. The interaction effect of Orientation and Agent Label was also not significant, F(1, 89) = 0.487, p = 0.487. In the context of our original d-prime results, which indicated a larger difference in recognition performance for upright versus inverted faces with the 'human' label compared to the 'android' label, this criterion analysis suggests that although participants' ability to discriminate was affected by these factors, their overall threshold for deciding whether to recognize a face was not swayed by the orientation or the label. This implies that the observed differences in d-prime are likely due to true perceptual discriminability rather than changes in decision bias.

## Discussion

Synthetic faces are increasingly realistic, to the point of near indistinguishability from actual human faces. However, such stimuli are not always processed in a fully face-like manner. In the present work, we sought to understand how believing that a face was human versus synthetic (i.e., non-human) would influence typical face perception processes. Specifically, we compared the extent to which human versus android labels affected face-typical processing in the face inversion task.

We found that when participants believed a face was that of an android, it elicited significantly smaller inversion effects than when they believed it was a human face. This was true even though all stimuli (i) were pictures of actual human faces and (ii) were randomly assigned to the human-versus-android labels, thus ruling out the possibility that the effects were driven by perceptual differences in the stimuli themselves. Indeed, while previous studies have demonstrated that visual differences between robot and human faces can affect performance in inversion tasks^56^ these findings cannot be fully explained by visual factors. Instead, it appears that the differences in how human and android faces are processed stem from the observer's beliefs rather than visual differences inherent to the stimuli.

Human-versus-android labeling muted but did not eliminate the effect of face inversion on recognition: even amongst ostensibly android stimuli there was still a robust effect of inversion on recognition performance. This makes sense given that all stimuli were actually human faces, and perceivers had significant prior expertise with this group of stimuli^57,58^. Even believing a human face to be non-human does not eliminate this prior perceptual experience, highlighting that person perception is best understood as a confluence of both top-down characteristics of perceivers (e.g., beliefs about targets; motives of perceivers) and bottom-up cues of the targets themselves (e.g., facial features; see^44^ for a review). Although we were able to influence face processing by altering participants' beliefs (top-down effects), the impact of face inversion on recognition, even when faces were labeled as androids, mean that actual human facial characteristics (bottom-up cues) still influence performance on face recognition tasks. This occurs despite believing that these faces are not human. This indicates that both the perceivers' top-down beliefs and the bottom-up visual features of the faces contribute to how we perceive and recognize individuals (see also^56^).

Beyond the implications for face perception, our results also have implications for research on human–robot interaction (HRI), mind perception (i.e., ascribing human-like traits to human and non-human agents^59^) and animacy (i.e., ascribing aliveness to human and non-human agents^60^), and social cognition research more broadly. Speaking first to HRI, the present work indicates that even highly human-like robots may not be fully perceived as social stimuli with emotions, identities and intentions, which has been suggested to influence various aspects of HRI, ranging from action understanding to acceptance^13,61–63^. Thus, robot designers should not focus solely on physical robot features, but also on factors outside of appearance to make robots appear “social”, such as behaviors or beliefs about non-human agents themselves (see^64^, for a review). Believing that an agent belongs to a social ingroup, or is similar to oneself in general, is an effective method of modulating perceptions of humanness. In HRI, it may be effective to emphasize similarities between a robot and an interacting human to boost face-like processing^59,65^. This could be accomplished via matching physical facial characteristics (e.g., eye color), behaviors (e.g., mirroring gestures) or personality characteristics (e.g., ways of speaking). Furthermore, previous research suggests that the mere belief a non-human entity has human-like capacities can cause perceivers to treat them as social entities, which could further influence face-like processing with non-human agents^66^. Thus, research in HRI would benefit from considering how such motives can influence the perception of robots.

The present research contributes to our understanding of how we perceive animacy and mental states in non-human entities. Research suggests that the extent to which stimuli are processed as mindless machines versus as mindful agents is modulated by perceivers’ motives for social connection. Past research demonstrates that humans’ innate desire to connect socially can motivate them to perceive intentionality in a robot in order satisfy this motivation^67,68^. Furthermore, when loneliness is induced in perceivers (i.e., via experimental manipulation), they are more likely to ascribe inner states and animacy to ambiguously animate agents (e.g., human-doll morphs^60^). This raises the possibility that such motives might modulate face perception processes in non-human agents as well. Perhaps when robots are designed for social interaction with human partners, this may motivate participants to attribute human-like traits to them, influencing how they perceive robot faces. Alternately, perhaps lonely perceivers might engage face-typical processing even for android robot faces (see^20^; for similar results with animals).

Our findings add to research examining the impact of top-down influences on early perceptual processes, such as face perception. However, the present work is distinct from other recent demonstrations in important ways. First, much recent research has focused more on social categories such as race and gender, and less on direct manipulations of the perceived humanness of agents. Second, whereas previous studies showed that inhumane behavior can lead to a decline in face-typical processing^47^), our research highlights that already beliefs about the non-human nature of a stimulus is sufficient to attenuate face perception. This is an important distinction as the capacity for inhumane acts is intrinsically linked to humanity; for instance, a lion hunting its prey is not considered inhumane, but a human harming another reflects a violation of human ethics. In our experiments, androids are treated as distinctly non-human rather than unethical or inhumane, highlighting that our findings are fundamentally different from previous findings due to the nature of the manipulation we employed. To our knowledge, our study is the first to find that believing a face lacks a human essence affects face-typical processing even when controlling for perceptual features of the stimuli. Although past work has hinted at this possibility^69,70^, the present work is the first that has manipulated the essential humanness of targets in a categorical manner.

The limitations of the current study provide avenues for future studies. First, we elected to manipulate the apparent humanness of faces via a human-versus-android label manipulation. This was intentional because we believed that the distinction between humans and androids would be plausible to some participants, while holding both the percept of the agents and some apparent mental capacities of the agents constant. For example, although androids are seen as lacking experiential capacities (i.e., emotional depth), both androids and humans are typically seen as sharing the capacity for mental agency (i.e., the ability to act on the world). However, highly realistic non-human faces generated via artificial intelligence could instead vary more widely in terms of their perceived capacities. For example, deepfakes may be seen as lacking minds altogether (i.e., experiential and agential capacities; e.g.,^71^) whereas androids have the ability to remember stimuli and to act on the world. Future research would benefit from understanding how inducing perceptions of different capacities in non-human stimuli may influence face perception.

Second, while it is clear that participants engaged in face-typical processing less strongly when believing stimuli to be androids, it is unclear where in the cognitive stream this took effect. Indeed, top-down effects on perceptual processing can take place at several levels, such as (i) the user’s focus of attention, (ii) which percepts are selected for further perceptual processing, (iii) perceptual organization of selected percepts (i.e., configural processing), and (iv) the representation of stimuli at a higher cognitive level^72^. Effects on all these levels could have manifested in smaller inversion decrements for android- versus human-believed stimuli. Since it is unclear from the current study what level of perception caused these differences in processing, future research would benefit from unpacking the temporal dynamics of these effects.

Third, while we interpret our findings within the context of embodied robotic agents, our study only examined static images. This limitation raises the question of whether the reported findings apply exclusively to static images and might not hold for physical robots. Indeed, past work has shown that static face images can have different effects than dynamic facial images, especially in situations where beliefs about the minds of targets (i.e., human versus robot) are being manipulated (e.g.^73^). Future research should explore whether embodied agents are also subject to disengagements in face-typical processing that occurs due to beliefs about targets.

Finally, we employed the face inversion paradigm to index face-typical processing. Although it is well established that faces are more sensitive to inversion than are most other objects, the inversion paradigm does not bear directly on the question of whether faces are processed in a qualitatively different manner than non-face stimuli^18^. Further, inversion could affect both perceptual processing broadly and face-like processing specifically. However, given that past work has shown that faces are more strongly affected by inversion than are most non-face objects, and given that we observe human-labeled faces were more strongly affected by inversion than were android-labeled faces, it seems reasonable to conclude that the android-label did reduce face-typical processing. However, it will be important in future research to compare inversion to other manipulations that affect image processing but not face orientation to differentiate where in the perceptual-cognitive stream that these effects emerge.

## Conclusion

In the present study, we find that manipulating beliefs about whether a face represents a real human versus a synthetic android influences face-typical processing, even when holding constant the faces themselves. To our knowledge, this is the first demonstration that believing a face represents a human or a robot, holding the stimulus itself constant, influences face perception. This research has implications not just for our understanding of face perception, but also for research in HRI, mind perception, and social cognition.

## Data Availability

The datasets used and/or analyzed during the current study available from the corresponding author on request.
